# Statistical Analysis of Haralick Texture Features to Discriminate Lung Abnormalities

**DOI:** 10.1155/2015/267807

**Published:** 2015-10-08

**Authors:** Nourhan Zayed, Heba A. Elnemr

**Affiliations:** Computer & Systems Department, Electronics Research Institute, Cairo 12611, Egypt

## Abstract

The Haralick texture features are a well-known mathematical method to detect the lung abnormalities and give the opportunity to the physician to localize the abnormality tissue type, either lung tumor or pulmonary edema. In this paper, statistical evaluation of the different features will represent the reported performance of the proposed method. Thirty-seven patients CT datasets with either lung tumor or pulmonary edema were included in this study. The CT images are first preprocessed for noise reduction and image enhancement, followed by segmentation techniques to segment the lungs, and finally Haralick texture features to detect the type of the abnormality within the lungs. In spite of the presence of low contrast and high noise in images, the proposed algorithms introduce promising results in detecting the abnormality of lungs in most of the patients in comparison with the normal and suggest that some of the features are significantly recommended than others.

## 1. Introduction

The lung is an organ that performs a multitude of vital functions every second of our lives. This fact leads to considering lung abnormalities, life-sustained diseases that have high priority in detection, diagnosis, and treatment if possible. Our focus in this paper will be on two popular abnormalities within the lung, which are pulmonary edema and lung tumor. Pulmonary edema (water in the lungs) is caused by fluid building up in the air sacs of the lungs [1, 2]. On the other hand, lung cancer/tumor is a disease where uncontrolled cell growth in tissues of the lung occurred [3].

Computer-aided diagnosis (CAD) schemes for thoracic computed tomography (CT) are widely used to characterize, quantify, and detect numerous lung abnormalities, such as pulmonary edema and lung cancer [4, 5]. An accurate lung segmentation method is always a critical first step in these CAD schemes and can significantly improve the performance level of these schemes. Although manual or semiautomatic lung segmentation methods for CT images were used in some early CAD schemes [6–10], they are impractical for current CAD schemes because multidetector CT (MDCT) scanners can generate hundreds of CT slices for a patient. An automated method for lung segmentation is needed for MDCT. In addition, the eye identification/detection of the abnormality type (pulmonary edema or tumors) in computed tomography (CT) images is very difficult even for the experienced clinicians because of its variable shape along with low contrast and high noise associated with it. As the final stage of treating the lung cancer is surgical removal of the diseased lung, hence it is necessary to identify the cancer location, which can be useful before they plan for the surgery.

The aim of our work is to develop an automated novel texture analysis based method for the segmentation of the lungs and the detection of the abnormalities, whether pulmonary edema or lung tumor. Haralick's features based on the gray level cooccurrence matrix (GLCM) are applied to capture textural patterns in lung images. The objective of this work is the selection of the most discriminating and finding out the significant texture features that can differentiate between these two types of abnormalities, in comparison to normal.

Haralick features are statistical features that are computed over the entire image. These measurements are utilized to describe the overall texture of the image using measures such as entropy and sum of variance. Chaddad et al. propose an approach, based on Haralick's features, to detect and classify colon cancer cells. This work aimed to select the most discriminating parameters for cancer cells [11]. A study to investigate the feasibility of using Haralick features to discriminate between “cancer” and “normal” subimages within a patient is illustrated in [12].

In this paper, CT images are first preprocessed for noise reduction and image enhancement, followed by segmentation techniques, as the tools to segment the lungs, and finally Haralick texture features [13–15] are calculated. Statistical analysis is done to detect the most significant Haralick features that will characterize the type of the abnormality within the lungs. Despite the low contrast and high noise existence in the images, the proposed algorithms introduce promising results in detecting the abnormality of lungs in most of the patients in comparison with the normal.

## 2. Materials and Methods

This paper presents a new automatic lung cancer detection system based on Haralick texture features extracted from the slice of DICOM Lung CT images. The proposed system is accomplished in four stages: image preprocessing, lung image segmentation, feature extraction, and classification. Statistical analysis is used to obtain the best features for classification to differentiate between lung cancer patients, ordered edema patients, and control subjects. The following sections will describe in detail these stages. All image analyses were achieved without any knowledge of patient clinical characteristics or status.

### 2.1. Dataset

Patients with either a lung cancer tumor or pulmonary edema were encompassed in the study. This study included two datasets, the first dataset referred to the Radiology Department at New Elkasr ElAiny teaching hospital, University of Cairo. The other dataset was obtained from The Cancer Imaging Archive (TCIA) sponsored by the SPIE, NCI/NIH, AAPM, and the University of Chicago [16]. The two datasets of 532 CT images from 37 different patients were included. The images are 512 × 512 stored in DICOM format. For each lung CT image, we separate the left lung from the right lung automatically, and each separated lung is labeled as normal or edema/cancer based on the dataset information.

### 2.2. Preprocessing

The main goal of preprocessing is to improve the quality of an image as well as make it in a form suited for further processing by human or machine [17]. This is accomplished by enhancing the visual appearance of an image besides removing the irrelevant noise and unwanted parts in the background. The proposed enhancement process, which is based on combining filters and noise reduction techniques for pre- and postprocessing as well, is carried out applying histogram equalization (HE) [18–20] followed by Wiener filtering [21, 22].

**Figure 1:**
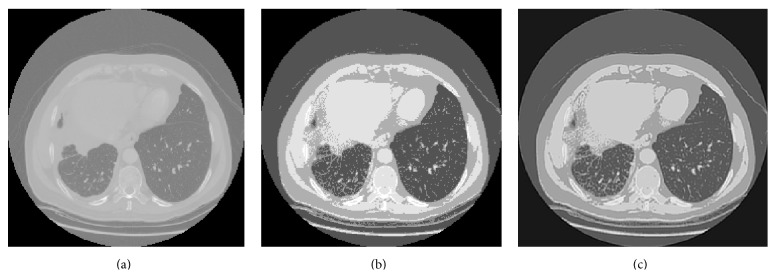
(a) The lung CT image; (b) the histogram equalized image; (c) the Weiner filtered output image.

Figure 1 presents the enhancement in the lung image contrast attained by applying the histogram equalization. However, the obtained gray scale image contains noises such as white noise and salt and pepper noise. Thus, Wiener filter is utilized to remove these noises from the enhanced lung image.  Figure 1(c) shows the effect of applying Weiner filter on the contrast enhanced lung image.

### 2.3. Lung Segmentation

**Figure 2:**
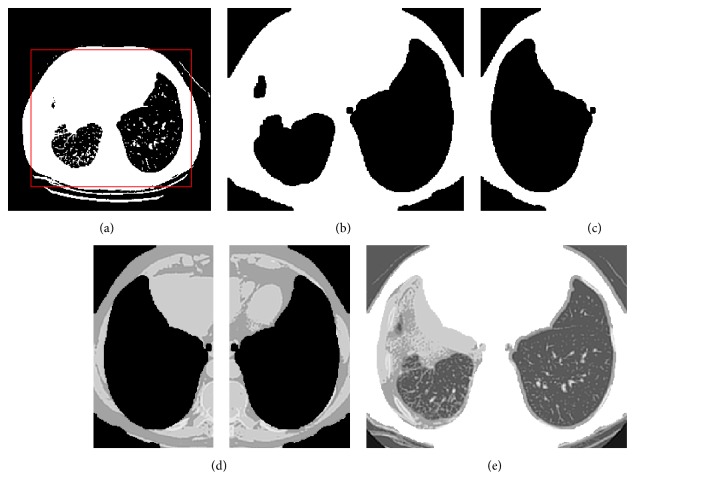
(a) The threshold image; (b) the eroded image; (c) the lung mask mirror; (d) the mask projection of the corresponding lungs images; (e) the extracted lungs.

Lung segmentation step aims to basically extract the voxels corresponding to the lung cavity in the axial CT scan slices from the surrounding lung anatomy. The segmentation technique proposed in [23] is utilized. This technique is based on the fact that there is a large density difference between air-filled lung tissues and surrounding tissues. Furthermore, both lungs are almost looking like mirror images of themselves in a human body. The segmentation of lung regions is achieved through the following steps. In the first step, the preprocessed CT image is converted into a binary image; a threshold of 128 was selected. Values greater than the threshold are mapped to white, while others less than that are marked as black. Consequently, the two lungs are marked and the area around them is cropped out. Second, an erosion morphological operation is employed in order to eliminate any white pixels within the two lungs. Afterward, the eroded and the original images are both divided into two equal regions. Black pixels for each region in the eroded image are counted; the region with the largest black area will be deemed as a lung mask. The attained lung mask is reflected in the opposite direction. As a result, right and left lung masks are obtained. These masks are multiplied with the corresponding original image regions; this will project the lung masks on the original two lungs images. Finally, update each black pixel in the obtained images by its original value; other pixels are set to 255.  Figure 2 illustrates the lung extraction process.

### 2.4. Feature Extraction

Feature extraction is the process of obtaining higher-level information of an image such as color, shape, and texture. Texture is a key component of human visual perception. Statistical texture methods analyze the spatial distribution of gray values, by computing local features at each point in the image and inferring a set of statistics from the distributions of the local features. Haralick et al. introduced Gray Level Cooccurrence Matrix (GLCM) and texture features back in 1973 [13]. This technique has been widely used in image analysis applications, especially in the biomedical field. It consists of two steps for feature extraction. The GLCM is computed in the first step, while the texture features based on the GLCM are calculated in the second step.

GLCM shows how often each gray level occurs at a pixel located at a fixed geometric position relative to each other pixel, as a function of the gray level [13]. The horizontal direction 00 with a range of 1 (nearest neighbor) was used in this work. The 9 texture descriptions used are presented in (4) to (13), where *N*_*g*_ is the number of gray levels, *p*_*d*_ is the normalized symmetric GLCM of dimension *N*_*g*_ × *N*_*g*_, and *p*_*d*_(*i*, *j*) is the (*i*, *j*)th element of the normalized GLCM [13].

Contrast (Moment 2 or standard deviation) is a measure of intensity or gray level variations between the reference pixel and its neighbor. Large contrast reflects large intensity differences in GLCM:

(1) $$\begin{array}{c} \text{Contrast}=\sum_{i}\sum_{j}{\left(\;i-j\;\right)}^{2}p_{d}\left(\;i,j\;\right). \end{array}$$

Homogeneity measures how close the distribution of elements in the GLCM is to the diagonal of GLCM. As homogeneity increases, the contrast, typically, decreases:

(2) $$\begin{array}{c} \text{Homogeneity}=\sum_{i}\sum_{j}\frac{1}{1+{\left(i-j\right)}^{2}}p_{d}\left(\;i,j\;\right). \end{array}$$

Entropy is the randomness or the degree of disorder present in the image. The value of entropy is the largest when all elements of the cooccurrence matrix are the same and small when elements are unequal:

(3) $$\begin{array}{c} \text{Entropy}=-\sum_{i}\sum_{j}p_{d}\left(\;i,j\;\right)\ln p_{d}\left(\;i,j\;\right). \end{array}$$

Energy is derived from the Angular Second Moment (ASM). The ASM measures the local uniformity of the gray levels. When pixels are very similar, the ASM value will be large. Consider

(4) Energy=ASMASM=∑i∑jpd2i,j.

Correlation feature shows the linear dependency of gray level values in the cooccurrence matrix:

(5) $$\begin{array}{c} \text{Correlation}=\sum_{i}\sum_{j}p_{d}\left(\;i,j\;\right)\frac{\left(i-\mu_{x}\right)\left(j-\mu_{y}\right)}{\sigma_{x}\sigma_{y}}, \end{array}$$

where *μ*_*x*_; *μ*_*y*_ and *σ*_*x*_; *σ*_*y*_ are the means and standard deviations and are expressed as

(6) μx=∑i∑jipdi,jμy=∑i∑jjpdi,jσx= ∑i∑ji−μx2pdi,jσy=∑i∑jj−μy2pdi,j.

The moments are the statistical expectation of certain power functions of a random variable and are characterized as follows.

Moment 1 (*m*_1_) is the mean which is the average of pixel values in an image and it is represented as

(7) $$\begin{array}{c} m_{1}=\sum_{i}\sum_{j}\left(\;i-j\;\right)p_{d}\left(\;i,j\;\right). \end{array}$$

Moment 2 (*m*_2_) is the standard deviation that can be denoted as

(8) $$\begin{array}{c} m_{2}=\sum_{i}\sum_{j}{\left(\;i-j\;\right)}^{2}p_{d}\left(\;i,j\;\right). \end{array}$$

Moment 3 (*m*_3_) measures the degree of asymmetry in the distribution and it is defined as

(9) $$\begin{array}{c} m_{3}=\sum_{i}\sum_{j}{\left(\;i-j\;\right)}^{3}p_{d}\left(\;i,j\;\right). \end{array}$$

And finally Moment 4 (*m*_4_) measures the relative peak or flatness of a distribution and is also known as kurtosis:

(10) $$\begin{array}{c} m_{4}=\sum_{i}\sum_{j}{\left(\;i-j\;\right)}^{4}p_{d}\left(\;i,j\;\right). \end{array}$$

Furthermore, difference statistics that are a subset of the cooccurrence matrix are also used. These features are based on the distribution of probability *P*_*x*−*y*_(*k*) which is defined as follows:

(11) $$\begin{array}{c} P_{x-y}\left(\;k\;\right)=\sum \sum C_{d}\left(\;i,j\;\right),\;k=0,1,\ldots ,N_{g}-1, \end{array}$$

where *C*_*d*_(*i*, *j*) is the (*i*, *j*)th element of the GLCM. The most basic difference statistic texture descriptions are the ASM, mean, and entropy:

(12) $$\begin{array}{c} \text{ASM}=\sum_{k}{\left(\;P_{x-y}\left(\;k\;\right)\;\right)}^{2}. \end{array}$$

When the *P*_*x*−*y*_(*k*) values are very similar or close, ASM is small. ASM is large when certain values are high and others are low:

(13) $$\begin{array}{c} \text{Mean}=\overset{}{\underset{k}{\sum }}kP_{x-y}\left(\;k\;\right). \end{array}$$

When *P*_*x*−*y*_(*k*) values are concentrated near the origin, mean is small and mean is large when they are far from the origin:

(14) $$\begin{array}{c} \text{Entropy}=-\sum_{k}P_{x-y}\left(\;k\;\right)\log P_{x-y}\left(\;k\;\right). \end{array}$$

Entropy is smallest when *P*_*x*−*y*_(*k*) values are unequal and largest when *P*_*x*−*y*_(*k*) values are equal.

The calculation of the Haralick texture features using the previous equations for the CT images volume sequences for every segmented lung (right and left) separately was performed. For each participant the gray level cooccurrence texture features: contrast, homogeneity, entropy, energy, correlation, and *m*_1_, *m*_2_, *m*_3_, and *m*_4_ accompanied by the difference statistical features: ASM, contrast, mean, and entropy were obtained for each segmented lung (right and left).

### 2.5. Statistical Analysis

For the purpose of random lung assignment in healthy volunteers, the left lung represented the diseased lung in the same percentage of cases as the patient population. For the acute data, two single factor analyses of variance (ANOVA) tests were conducted for each Haralick texture feature measurement between affected (either left or right) and fellow lung (either left or right) for both categories cancer and edema patients. A single factor analysis of variance (ANOVA) was conducted as well between patients and controls. Other between-subject single factor analyses were conducted to find out the significant Haralick features that could differentiate cancer from edema.

## 3. Experimental Results

Two datasets of 532 CT images were included. For each lung CT preprocessed image, we separate the left lung from the right lung automatically as discussed before in Section 2.3, and each separated lung is labeled as normal or edema/cancer based on the dataset information. The Haralick texture features measurements for each lung separately are calculated (the gray level cooccurrence texture features: contrast, homogeneity, entropy, energy, correlation, and moments along with the difference statistical features: ASM, mean, and entropy). The mean and the standard deviation of the Haralick texture features measurements calculated as well as the ANOVA results are given for tumor patients affected lung versus fellow lung in Table 1 and for pulmonary edema patients in Table 2. The ANOVA summary of statistics for either pulmonary edema or tumor patients versus normal is given in Table 3. The significant Haralick texture features that can differentiate between pulmonary edema and tumor are found in Table 4.

From Table 1, we can conclude that Haralick texture features measurements (homogeneity, energy, correlation, and entropy) of the affected cancer lung were significantly different than that of the fellow lung. The homogeneity, energy, and correlation were significantly less than those of the normal fellow lung. While entropy of the cancerous lung is approaching being significantly more than that of the fellow lung, Moment 3 and the difference statistical feature ASM (diff_ASM) texture feature measurement of the cancerous lung is approaching being significantly less than that of the normal lung.

Table 2 showed that Haralick texture features measurements (homogeneity, entropy, and moments calculated from the cooccurrence matrix as well as mean and ASM computed from the difference statistics) of the pulmonary edema affected lung were also significantly different than those of the control subject lung; moreover contrast and entropy computed from the difference statistics were significantly more than those of the fellow lung.

Considering Tables 1 and 2, we can conclude from Table 3 that the homogeneity, energy, entropy, *m*_3_, *m*_4_, diff_ASM, diff_mean, and diff_entropy are good biomarkers to significantly differentiate between diseased and normal lungs without any disease specification. On the other hand, the results illustrated in Tables 1, 2, and 4 show that entropy and the entropy calculated from the difference statistics would be a good candidate to significantly differentiate between pulmonary edema and cancer.

## 4. Conclusion and Discussion

The texture features analyses are well known approaches to quantify and express the heterogeneity that may not be appreciated by clinical naked eyes, and it was presented before as good imaging biomarkers to differentiate between diseases. In this paper an evaluation of the Haralick texture features is done in order to identify the most significant features that can be used in order to detect and differentiate abnormalities within the lungs for cancer and edema versus normal. Our results indicate that entropy determined by gray level cooccurrence matrix and ASM is significantly different in edema patients versus normal while it is not in cancer patients versus normal. Since the entropy is the degree of randomness or the degree of disorder in the image, and the angular second moment represents the uniformity in the image, this may be interpreted as the cancer disease causing a localized heterogeneity in the diseased specified area in the lung while the edema causes heterogeneous disorder in the whole lung image. High entropy values calculated implies that the elevated level of disorder and disorganization occurred due to the edema diseased lung versus the cancer diseased lung. The energy feature that is derived from the angular second moment measures and representing the local uniformity of the gray levels is a good biomarker to differentiate between cancer and edema diseases. From Table 2, contrast is a good biomarker for the pulmonary edema disease and this agrees with the texture feature meaning which means high contrast values for heavy texture changes. Gray level cooccurrence matrix textural properties such as homogeneity, correlation, mean, and moments are good significant biomarkers for diseased lung versus normal ones in general without any specification for the disease type. Our results agree with other articles indicating that textural analysis has the potential to develop into a valuable clinical tool that improves the diagnosis, tumor staging, and therapy assessment.

While our results are promising, there is still further work that can be done in the detecting of the abnormality within the lungs to detect the type of that abnormality whether it will be a lung cancer or edema. A preliminary investigation has been done using statistical analysis to identify the most useful texture features that can be fed to any classification technique later. This statistical analysis is done using ANOVA. After selecting these features we can feed them for better localization and classification as further work.

## Figures and Tables

**Table 1:** ANOVA (1 within-subject factor) results for cancer patients Haralick texture features (comparison between AL and FL). AL: affected lung; FL: fellow lung.

Feature name	AL (average ± SEM)	FL (average ± SEM)	AL versus FL
Homogeneity	0.511 ± 0.01	0.517 ± 0.01	**F**(1,426) = 22.0 **p** < 0.000004
Energy	0.372 ± 0.01	0.374 ± 0.01	**F**(1,426) = 15.1 **p** < 0.0001
Correlation	0.964 ± 0.001	0.965 ± 0.001	**F**(1,426) = 6.15 **p** < 0.013
Contrast	231.98 ± 4.54	231.76 ± 4.54	*F*(1,426) = 0.012 *p* < 0.911
Entropy	8.0 ± 0.19	7.94 ± 0.19	**F**(1,426) = 11.8 **p** < 0.0007
*m*_1_	0.003 ± 0.02	0.007 ± 0.02	*F*(1,426) = 0.029 *p* < 0.88
*m*_2_	231.13 ± 4.54	231.75 ± 4.01	*F*(1,426) = 0.012 *p* < 0.911
*m*_3_	−164 ± 190.79	−683.99 ± 155.33	*F*(1,426) = 2.65 *p* < 0.09
*m*_4_	1784467 ± 83311	1654941 ± 56455	*F*(1,426) = 6.25 *p* < 0.19
Diff_ASM	0.226963389 ± 0.006	0.229353096 ± 0.005	*F*(1,426) = 3.18 *p* < 0.06
Diff_Mean	6.195 ± 0.08	6.28 ± 0.09	*F*(1,426) = 2.16 *p* < 0.12
Diff_Entropy	3.159 ± 0.03	3.55 ± 0.03	*F*(1,426) = 2.32 *p* < 0.10

**Table 2:** ANOVA (1 within-subject factor) results for edema patients Haralick texture features (comparison between AL and FL). AL: affected lung; FL: fellow lung.

Feature name	AL (average ± SEM)	FL (average ± SEM)	AL versus FL
Homogeneity	0.64 ± 0.013	0.60 ± 0.020	*F*(1,105) = 2.16 *p* < 0.15
Energy	0.428 ± 0.01	0.429.01 ± 0.01	*F*(1,105) = 0.029 *p* < 0.87
Correlation	0.006 ± 0.001	0.008 ± 0.001	*F*(1,105) = 2.32 *p* < 0.141
Contrast	177.07 ± 5.89	188.58 ± 4.26	**F**(1,105) = 15.1 **p** < 0.0002
Entropy	2.10 ± 0.04	2.19 ± 0.067	**F**(1,105) = 1.28 **p** < 0.269
*m*_1_	0.52 ± 0.03	−0.47 ± 0.02	**F**(1,105) = 41.8 **p** < 0.000001
*m*_2_	199.975 ± 9.658	218.583 ± 10.085	*F*(1,105) = 2.20 *p* < 0.152
*m*_3_	5219 ± 1436	−7539 ± 885	**F**(1,105) = 41.8 **p** < 0.000001
*m*_4_	2854294 ± 208886	2382237 ± 263250	*F*(1,105) = 2.12 *p* < 0.158
Diff_ASM	0.377 ± 0.01	0.288 ± 0.08	**F**(1,105) = 4.56 **p** < 0.043
Diff_Mean	4.07 ± 0.4379	4.89 ± 0.48478	**F**(1,105) = 7.87 **p** < 0.01
Diff_Entropy	2.96 ± 0.05	3.29 ± 0.05	**F**(1,105) = 4.73 **p** < 0.039

**Table 3:** ANOVA (1 within-subject factor) results summary of statistics *p* value for patients (either edema or cancer) Haralick texture features versus normal controls.

Feature name	Diseased versus normal controls (*p* value)	Feature name	Diseased versus normal controls (*p* value)
Homogeneity	**p** < 0.00002	*m*_2_	*p* < 0.229
Energy	**p** < 0.0006	*m*_3_	**p** < 0.0002
Correlation	*p* < 0.485	*m*_4_	**p** < 0.002
Contrast	*p* < 0.229	Diff_ASM	**p** < 0.000001
Entropy	**p** < 0.0004	Diff_Mean	**p** < 0.000005
*m*_1_	**p** < 0.0007	Diff_Entropy	**p** < 0.0004

**Table 4:** ANOVA (1 between-subject factor) results summary of statistics *p* value for patients Haralick texture features cancer versus edema patients.

Feature name	Cancer versus edema patients (*p* value)	Feature name	Cancer versus edema patients (*p* value)
Homogeneity	**p** < 0.0002	*m*_2_	*p* < 0.69
Energy	*p* < 0.065	*m*_3_	**p** < 0.01
Correlation	*p* < 0.179	*m*_4_	*p* < 0.89
Contrast	*p* < 0.699	ASM	*p* < 0.73
Entropy	**p** < 0.017	Mean	**p** < 0.032
*m*_1_	**p** < 0.0004	Entropy	**p** < 0.007
